# Characterizing emotional Stroop interference in posttraumatic stress disorder, major depression and anxiety disorders: A systematic review and meta-analysis

**DOI:** 10.1371/journal.pone.0214998

**Published:** 2019-04-09

**Authors:** Marilyne Joyal, Tobias Wensing, Jean Levasseur-Moreau, Jean Leblond, Alexander T. Sack, Shirley Fecteau

**Affiliations:** 1 Medical School, Laval University, Quebec City, QC, Canada; 2 Centre intégré universitaire en santé et services sociaux de la Capitale-Nationale, Quebec City, QC, Canada; 3 Faculty of Psychology and Neuroscience, Maastricht Brain Imaging Center, Maastricht University, Maastricht, The Netherlands; Yale University, UNITED STATES

## Abstract

**Background:**

Posttraumatic stress disorder is a debilitating psychiatric disorder characterized by symptoms of intrusive re-experiencing of trauma, avoidance and hyper-arousal. Diagnosis and treatment of PTSD is further complicated by concurrently occurring disorders, the most frequent being major depressive disorder and anxiety disorders. Previous research highlights that attentional processing in posttraumatic stress disorder is associated with substantial interference by emotional stimuli, a phenomenon also observed in these concurrently occurring psychiatric disorders. However, the diagnosis-relevance of this interference remains elusive. Here, we investigated the emotional Stroop interference for diagnosis-related stimuli, generally negative stimuli, and generally positive stimuli in posttraumatic stress disorder, major depressive disorder and anxiety disorders.

**Methods:**

We performed a systematic database search in PubMed (Medline), Cochrane Library and PsycINFO on emotional Stroop performance in individuals with a diagnosis of posttraumatic stress disorder, major depressive disorder or anxiety disorders separately. Mean effect sizes, standard errors and confidence intervals were estimated for each clinical group and healthy control group comparison using random effect models.

**Results:**

As compared to healthy control group, the posttraumatic stress disorder group displayed greater interference by diagnosis-related stimuli and positive stimuli but not for generally negative stimuli. The major depressive disorder and anxiety disorders groups showed greater interference by diagnosis-related and negative stimuli, but not by positive stimuli. The age and sex had no significant impact on interference.

**Conclusions:**

These findings highlight the importance of diagnosis-relevant information on attentional processing in all three clinical populations, posttraumatic stress disorder, major depressive disorder and anxiety disorders. Further, the impact of generally negative stimuli but not generally positive stimuli in major depressive disorder and anxiety disorders indicate impaired attentional bias for mood-congruent stimuli but not for general stimuli. Finally, it remains to be studied whether the influence of generally positive stimuli in posttraumatic stress disorder indicate that positive stimuli are perceived as PTSD related.

## Introduction

Individuals diagnosed with posttraumatic stress disorder (PTSD) have been exposed to a traumatic event comprising physical or psychological harm. PTSD is a highly debilitating disorder defined by the direct or indirect exposure to a traumatic event, including symptoms of intrusive re-experience, trauma-related thoughts and feelings and hypervigilance [1]. Hence, PTSD can lead to considerable impairments in individual well-being, from everyday functioning up to suicidal ideation and suicide attempts [2–4].

Symptom severity in PTSD and symptoms of re-experiencing, hypervigilance, avoidance and levels of anxiety have been positively correlated with attentional bias [5,6]. Attentional bias in PTSD usually refers to persistent engagement of attentional processing mechanisms to real or experimentally evoked threatening events. Indeed, attentional processing in PTSD is associated with interference by emotional stimuli [6–17]. Hence, recent clinical trials implemented the existing knowledge on the mechanisms of attentional bias in PTSD into a novel treatment approach, that is, attentional bias modification (ABM) [18,19]. ABM utilizes the detection-of-object (dot) probe task to elicit attentional bias. Studies on ABM treatment reported a decrease in PTSD symptoms with effect sizes similar to those of placebo-pill pharmacotherapy outcomes. Furthermore, ABM treatment did not improve attentional bias [20], even when the ABM approach was tailored to personally relevant bias-evoking stimuli [21]. Given the lack of evidence for ABM as a treatment for PTSD, there is a need to further understand attentional bias in PTSD.

The Stroop task [22] is a well-established cognitive task to investigate attentional processing of simultaneously occurring sensory information in context of selective attention, cognitive set shifting and response inhibition (see for example MacLeod or Stein et al.) [23,24]. In the original version of the color-word Stroop task (CW-Stroop) [25], participants are presented with three different card templates: the first template contains black-on-white printed words of highly distinguishable colors (“blue”, “green”, “red”, “yellow”); the second template depicts rectangles printed in blue, green, red or yellow color (“color-naming” condition); and the last template shows the same words as the first template but printed in a color that does not represent its actual content (“incongruent” condition; e.g., the word “blue” printed in green). Performance on the first template serves as a measure of reading abilities. Then, a Stroop interference index is calculated by subtracting response times (RT) in the color-naming condition from those in the incongruent condition. This index serves as a measure of the attentional engagement and hence the attentional bias. Nowadays, computerized versions of the Stroop paradigm allow for specific modifications in experimental designs that serve individual study purposes (e.g., stimulus presentation in a trial-by-trial fashion instead of a blocked design using physical template cards).

The emotional Stroop task modifies the CW-Stroop rationale by replacing color-words with neutral and emotionally loaded stimuli (e.g., the word “violence” painted in blue compared to a neutral word in the same color) [26]. In healthy participants, the emotional Stroop task does not elicit attentional bias related to threatening stimuli when stimuli were presented in a single-item fashion (i.e., one stimulus at a time) [18]. Individuals diagnosed with PTSD, however, often display attentional bias for negative stimuli as compared to healthy controls at the emotional Stroop task (i.e., threatening or aversive) [9,13]. Hence, emotional interference appears to be related to the thematic relevance of the personally experienced event and not merely on the exposure to threat *per se* in both civilians [15] and military personnel [5]. In survivors of sexual violence, for instance, an attentional bias was observed for both intimacy-related trauma words (e.g., “rape”) and intimacy-related positive words (e.g., “love”) [5]. However, recent findings on emotional interference, especially in military personnel with acute PTSD, challenge the view of an attentional bias for threatening or trauma-related information. These studies report an attentional shift away from threatening information, suggesting an avoidance pattern [19,27,28]. Given these inconsistencies, a meta-analysis aimed to synthesize findings on emotional Stroop task performance in PTSD. This meta-analysis indicated that individuals with PTSD, as compared to healthy controls, displayed impairments in the emotional Stroop task when processing trauma-related and generally threatening, but not positive information [29].

Attentional bias is also important in patients with major depressive disorder (MDD) or anxiety disorders (AD). During neuropsychological tests, individuals diagnosed with MDD and AD also display abnormal attentional bias to emotionally loaded stimuli [18,30]. This is of particular importance because MDD and AD are among the most concurrent diagnoses in PTSD. Further, these concurrent psychiatric disorders greatly impact and hamper diagnosis and treatment of PTSD [31–33]. For instance, co-occurrence of PTSD and MDD symptoms considerably elevates risk for suicidal ideations and suicide attempts compared to the impact of each disorder on its own [34–37].

The aim of this work is thus to investigate attentional bias in individuals diagnosed with PTSD, MDD and / or AD. Hence, we systematically reviewed findings on the emotional Stroop paradigm addressing task performance in individuals with PTSD, MDD, and /or AD, as compared to healthy controls. We used meta-analytic statistical tools to assess whether interference by stimuli with emotional valence in these groups can be attributed to non-specific emotional valence (generally positive or negative) or diagnosis-relevant emotional words.

## Materials and methods

### Methodological quality assessment

We conducted this review following PRISMA guidelines [38]. The PRISMA checklist is provided in supplementary material (S1 Table).

### Search strategy

We conducted a systematic database search in PubMed (Medline), Cochrane Library and PsycINFO on peer-reviewed articles published between 1986 and 30 April 2018, using combinations of the search strings “posttraumatic stress disorder (PTSD)” or “major depressive disorder (MDD)” or “anxiety disorder (AD)” and “modified/emotional Stroop task”. Detailed search terms are provided in supplementary material (S2 Fig). We limited our search to published peer-reviewed articles as they tend to show higher methodological quality than unpublished studies [39–40]. In the field of psychiatry research, inclusion of grey literature may also increase risk of bias [41]. We applied a filter to identify relevant literature examining humans only. This database search resulted in a total of 900 abstracts for potential inclusion.

### Selection criteria

**Fig 1:**
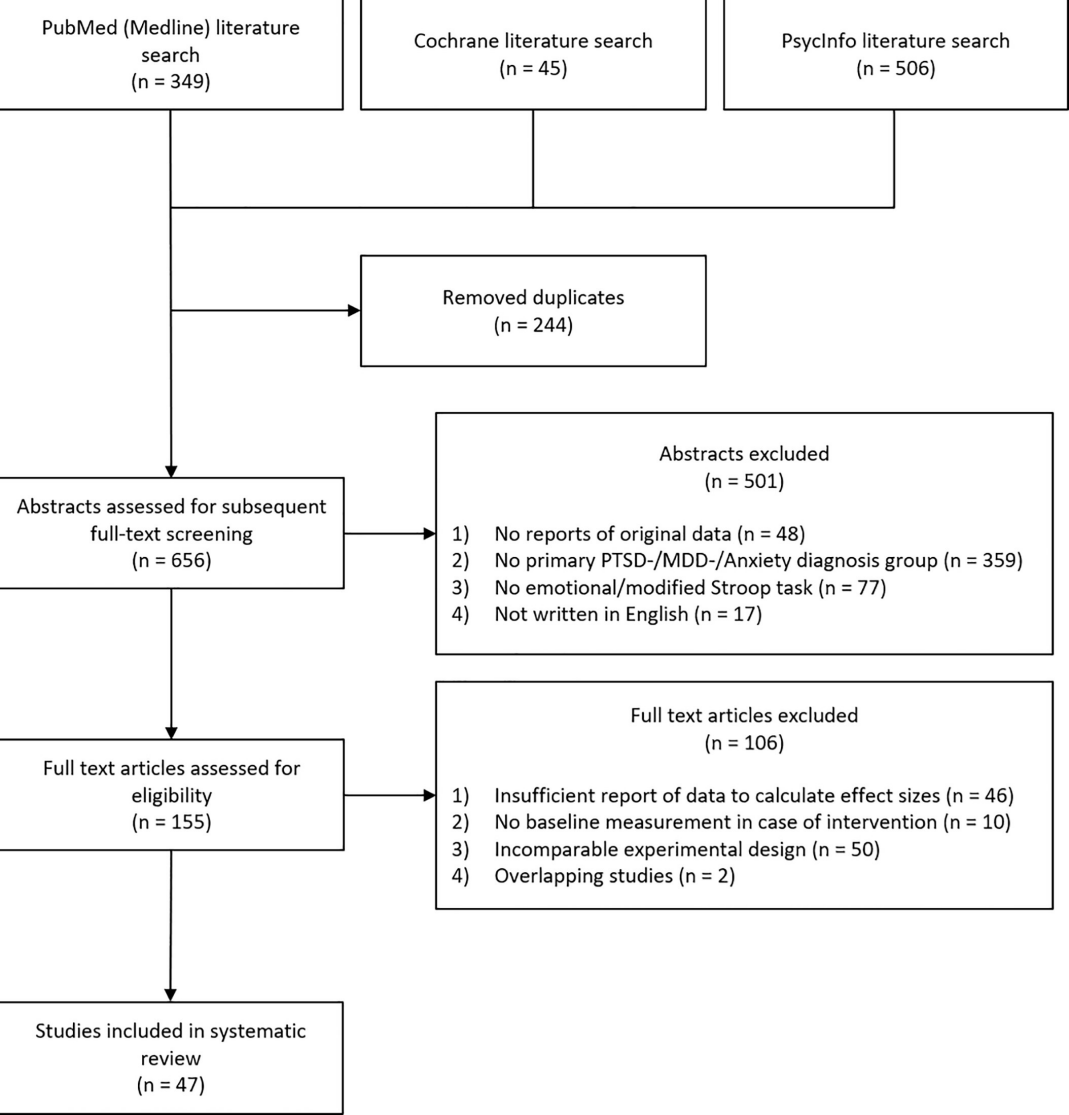
Schematic overview of the meta-analytic study selection procedure in this review. PTSD = Posttraumatic Stress Disorder, MDD = Major Depressive Disorder.

In a first phase of selection, we excluded abstracts (a) not reporting original data (e.g., reviews, meta-analyses, opinion letters), (b) not assessing a group with a primary diagnosis of PTSD, MDD, or AD, (c) not implementing the modified or emotional Stroop task as a cognitive measurement, and (d) not written in English, leaving a total of 155 original studies. In order to conduct coherent statistical analyses, we further excluded 108 of the 155 studies due to the following reasons: (a) no sufficient behavioral data to calculate effect sizes (neither interference scores nor response times to emotional and neutral conditions), (b) experimental manipulation prior to baseline behavioral testing (e.g., assessment of treatment efficacy), (c) experimental designs that were incomparable to designs of the remaining studies (i.e. stimulus presentation in a multi-item instead of a single-item/trial-by-trial design, stroop task with masked presentation mode or with pictures instead of written words) and (d) overlapping studies. Based on the procedure described above, 47 studies [5–7,42–85] were included in statistical analyses (54 datasets: PTSD: 13; MDD: 12; AD: 29) (Fig 1).

### Data extraction

Data extraction was performed by two independent reviewers (TW, JLM). Differences regarding outcome variables were resolved by consensus.

#### Qualitative data

We collected demographics and experimental design characteristics from all studies including, if available, (a) study type, (b) clinical diagnostics, (c) number of participants, (d) mean age, (e) female/male ratio, (f) medication, (g) comorbidity status, (h) stimulus valence, and (i) response modality. We further assessed potential risk of bias in included studies using the Cochrane risk of bias tool [86]. The Cochrane risk of bias tool allows for inference of studies’ quality via an observer-dependent rating system that scores potential methodological shortcomings as “low”, “high” or “unclear” (S2 Table).

#### Quantitative data

We extracted mean response times (RT), mean interference scores, as well as respective dispersion indices (Standard Deviation [SD] and Standard Error [SE]) from all studies.

### Outcome measures of the emotional Stroop task

Within the emotional Stroop task, participants are asked to identify the color of either neutral or emotionally loaded stimuli while ignoring its actual content. Interference by a specific type of stimulus is calculated by RT difference scores (i.e., subtracting mean RT of the neutral stimuli from that of emotional stimuli). In this regard, positive values mirror longer RT for emotionally loaded stimuli and negative values indicate shorter RT for emotionally loaded stimuli (see for example El Khoury-Malhame et al.) [13]. Interference scores were our primary outcome measure, calculated on RT measurements. Across studies, RT was commonly defined as the latency from stimulus onset to the participant’s response. RTs of the Stroop tasks were reported in milliseconds (ms) and accompanied by statistical dispersion indices (SD, SE).

### Stimulus type and response modalities

Stimuli consisted of words and were presented in a trial-by-trial fashion (i.e., one stimulus at a time) in a randomized or quasi-randomized order on a computer monitor. Participants were instructed to respond verbally (e.g., recorded by microphone on a computer) or by pressing a button that was specific to the color of the displayed word. Twenty-nine group comparisons measured RTs of verbal responses (PTSD: n = 8; MDD: n = 6; AD: n = 15) and 25 group comparisons measured RTs by button press (PTSD: n = 5; MDD: n = 6; AD: n = 14).

### Arousal ratings and stimulus valence

This section describes how word stimuli were categorized in the studies included in this review. Ratings of stimuli valence and/or arousal were based on participants’ self-reports prior to or after testing (4 studies), ratings in pilot experiments with independent cohorts of participants (6 studies), evaluations by clinicians or otherwise trained professionals (8 studies) or were taken from previous research and validated stimuli databases (27 studies). In four studies, assessment of arousal or stimulus rating was not further specified. Based on stimulus ratings, and for the sake of comparability across studies and psychiatric diagnostic groups, word stimuli included in the present analysis were categorized as (a) diagnosis-relevant (i.e. PTSD-relevant, e.g. war, abuse; MDD-relevant, e.g. sadness, discouraged; AD-relevant, e.g. panic, embarrassed) (b) generally negative (e.g. fraud, divorce), or (c) generally positive (e.g. pleasant, comedy). Diagnosis-relevant cues had the distinction of being related to the trauma inducing-event or to the symptomatology of clinical groups. In all studies, stimuli with neutral valence were utilized for calculation of interference scores.

### Statistical data analysis

Statistical analyses were separately performed for each of the three clinical groups (PTSD-groups, MDD-groups, AD-groups). Effect sizes (bias-corrected standardized mean-differences; Hedge’s g) were calculated with the R *Metafor* package (R 3.5.0; R Development Core Team) for each study contrasting clinical and healthy control groups. Single-study effect sizes were analyzed using meta-analytic random-effect models and the Hedge’s g estimator to derive a meta-analytic overall effect size (*g*) and tests for heterogeneity (Q and I^2^) for each group comparison of interest. We also explored whether some variables had an impact on interference using moderator analyses. There were sufficient available data to test mean age of patients, percentage of women in clinical groups, and response modality at the emotional Stroop task. Separate models were fitted to determine the main effects of each variable. In case of large heterogeneity across studies, outliers were identified based on visual inspection of the meta-analytic forest plots. To further test whether any one study was overly influential on effect size estimates, we conducted leave-one-out cross-validation for these group comparisons of interest.

## Results

### Study types

Case-control studies was the most common study type. For the PTSD groups, articles described case-control studies (11 studies), controlled before-after study (1 study), and randomized controlled trial (1 study). All articles with MDD groups described case-control studies (12 studies). For AD groups, study types were case-control studies (21 studies), controlled before-after study (1 study), randomized controlled trial (1 study), and crossover study (1 study). One article with AD patients also included three experiments, with both case control and before-after designs. In all cases, we extracted data from the baseline condition to avoid any experimental manipulation prior to behavioral testing.

### Study groups

#### Clinical groups

Clinical groups comprised individuals with a primary diagnosis of either (a) PTSD, (b) MDD or (c) AD according to administered clinical interviews and scales (Tables 1–3). Diagnoses of AD included generalized anxiety disorder—not otherwise specified (GAD NOS), panic disorder (PD), social phobia (SoP), specific phobia (SpP) and multiple diagnoses of AD within one study group. To enable statistical group comparisons of interest and provide an overall view of attentional bias in AD in general, all differential AD diagnoses were summarized in a single AD group. In each individual study, clinical assessments were based on the DSM [1] or the International Classification of Disorders (ICD) [87] using the most recent version at the respective time-point of assessment. Across studies, 288 participants were considered in the PTSD-groups, 243 participants in the MDD-groups and 613 participants in the AD-groups.

**Table 1:** Characteristics of included studies with posttraumatic stress disorder (PTSD) groups.

Reference	Study type^A^	Clinical Scale^B^	N of subject (clinical; HC)	Age (mean ± SD)	Female patients (%)	Rx	Comorbidity	Stimulus valence^C^	Response modality
Ashley[5]	CC	CPRS	30; 30	32.2 ± 7.9	3.3	-	Yes	GN, GP, PTSD	Verbal
Buckley[42]	CC	SCID	6; 6	34.7 ± 7.0	100	-	-	PTSD	Verbal
Cassiday[43]	CC	SCID	12; 12	33.2 ± 10.3	91.7	Yes, not specified	-	GN, GP	Verbal
Harvey[44]	CC	PTSD-I	20; 20	34.0 ± 10.9	70.0	-	-	PTSD	Verbal
Herzog[45]	CC	SCID, CAPS	28; 28	30.6 ± 10.0	100	Yes	Yes	GN, PTSD	Motor
Khanna[46]	CC	CAPS	26; 16	33.9 ± 9.0	0.0	-	Yes	GN, PTSD	Verbal
El Khoury-Malhame[6]	CBA	MINI	19; 19	45.0 ± 15.0	63.2	Yes	Yes	GN	Motor
Martinson[47]	CC	SCID	33; 35	23.6	78.8	-	-	PTSD	Motor
McNally[48]	CC	CMISS	15; 12	48.0 ± 13.4	100	-	-	GP, PTSD	Verbal
Metzger[49]	CC	SCID	9; 10	37.0 ± 10.0	66.7	No	Yes	GP, PTSD	Motor
Paunovic[7]	CC	CAPS	39; 39	35.7 ± 9.7	39.3	Yes	Yes	GP, PTSD	Verbal
Thomaes[50]	RCT	CAPS	29; 22	33.5 ± 11.6	100	Yes	Yes	GN, PTSD	Motor
Wittekind[51]	CC	SCID	22; 11	71.0 ± 2.4	68.2	-	-	GN, PTSD	Verbal

^A^ CBA = Controlled before and after study, CC = Case-control, RCT = Randomized Controlled Trial

^B^ CPRS = Computerized Patient Record System, SCID = Structured Clinical Interview for DSM, PTSD-I = PTSD-Interview, CAPS = Clinician Administered PTSD Scale, MINI = Mini-Internal Neuropsychiatric Interview for DSM, CMISS = Mississippi Scale for Combat-Related Posttraumatic Stress Disorder (civilian version)

^C^ GN = Generally negative, GP = Generally positive, PTSD = Posttraumatic stress disorder-relevant; Rx = Prescriptions

**Table 2:** Characteristics of included studies with major depressive disorder (MDD) groups.

Reference	Study type^A^	Clinical Scale^B^	N of subject (clinical; HC)	Age (mean ± SD)	Female patients (%)	Rx	Comorbidity	Stimulus valence^C^	Response modality
Broomfield[52]	CC	GDS	16; 19	73.0 ± 6.2	56.3	Yes	-	GN, GP	Motor
Constant[53]	CC	SCID	20; 26	47.7	60.0	Yes	-	MDD	Verbal
Dozois[54]	CC	SCID	24; 25	38.8 ± 12.7	-	Yes	-	GN, GP	Verbal
Fritzsche[55]	CC	SCID	20; 20	40.6 ± 9.2	50.0	Yes	-	GP, MDD	Verbal
Gupta[56]	CC	SCID	10; 10	40.0 ± 8.4	50.0	Yes	No	GN, GP	Motor
Lim[57]	CC	ADIS	33; 33	39.7 ± 8.8	-	Yes	-	GN, GP, MDD	Verbal
Markela-Lerenc[58]	CC	SCID	23; 27	41.0 ± 11.4	47.8	Yes	-	GP, MDD	Motor
Matsubara[59]	CC	DSM-IV-TR	16; 20	45.4 ± 2.2	50.0	Yes	No	GN, GP, MDD	Motor
McNeely[60]	CC	SCID	15; 14	38.5 ± 8.7	73.3	Yes	Yes	GN, GP	Motor
Mitterschiffthaler[61]	CC	SCID	17; 17	39.3 ± 9.4	82.4	No	-	MDD	Verbal
Mogg[62]	CC	DSM-III-R	18; 18	34.8	77.8	-	-	GP, MDD	Verbal
Schlosser[63]	CC	SCID	31; 37	38.7 ± 10.4	48.4	Yes	Yes	GN, MDD	Motor

^A^ CC = Case-control

^B^ GDS = Geriatric Depression Scale, SCID = Structured Clinical Interview for DSM, ADIS = Anxiety Disorders Interview Schedule, DSM-IV-TR = Diagnostic Manual of Mental Disorders (4^th^ Edition, text-revision), DSM-III-R = Diagnostic Manual for DSM Disorders (3^rd^ Edition, revision)

^C^ GN = Generally negative, GP = Generally positive, MDD = Major depressive disorder-relevant; Rx = Prescriptions

**Table 3:** Characteristics of included studies with anxiety disorder (AD) groups.

Reference	Study type^A^	Clinical Scale^B^	N of subject (clinical; HC)	Age (mean ± SD)	Female patients (%)	Rx	Comorbidity	Stimulus valence^C^	Response modality
***A) Generalized anxiety disorder (GAD NOS)***									
Bradley[64]	CC	DSM-III-R	20; 11	37.8 ± 11.3	40.0	-	Yes	GAD	Verbal
Chen[65]	CC	DSM-IV-TR	42; 26	34.3 ± 8.0	54.8	Yes	-	GAD, GP	Motor
Dozois[54]	CC	SCID	25; 25	38.9 ± 10.4	-	Yes	-	GN, GP	Verbal
Mogg[62]	CC	DSM-III-R	19; 18	38.2	73.7	-	-	GAD, GP	Verbal
Price[66]	CC	SCID	16; 12	63.1 ± 3.1	68.8	Yes, not specified	Yes	GN	Motor
***B) Panic disorder (PD)***									
Chen[65]	CC	DSM-IV-TR	34; 46	31.6 ± 8.6	67.7	Yes	-	GP, PD	Motor
De Cort[67]	CC	DSM-IV	32; 30	42.5 ± 12.3	53.1	-	-	GN, PD	Motor
Deppermann[68]	RCT	DSM-IV-TR	G1: 14; 19 G2: 12; 19	38.4 39.1	50 75	Yes Yes	Yes Yes	PD	Motor
Dresler[69]	CC	SCID	20; 23	31.7 ± 7.4	55.0	Yes	Yes	PD	Motor
Dresler[70]	CC	SCID	17; 26	40.0 ± 11.6	58.8	Yes	Yes	PD	Motor
Gropalis[71]	CBA	SCID	25; 31	31.1 ± 8.1	64.0	-	Yes	PD	Motor
Kampman[72]	Exp 1–2: CC Exp 3: before after study	DSM-IV	18; 18	38.9 ± 12.6	-	-	-	GN, PD	Verbal
Lim[57]	CC	ADIS	33; 33	39.7 ± 8.8	-	Yes	-	GN, GP	Verbal
Lundh[73]	CC	ADIS	35; 35	37.2 ± 9.4	71.4	Yes, partially specified	Yes	GN, PD	Verbal
Maidenberg[74]	CC	ADIS-R	15; 15	Range: 21–49	-	No	-	GN, GP, PD	Verbal
McNally[75]	CC	ADIS-R	14; 14	36.6	64.3	-	-	GN	Verbal
McNally[76]	CC	SCID	24; 24	32.0 ± 9.0	91.7	-	-	GN, GP	Verbal
McNally[77]	CC	SCID	16; 16	-	93.7	-	-	GN, GP, PD	Verbal
Reinecke[78]	CC	SCID	23; 22	28.6 ± 8.1	69.6	No	Yes	GN, PD	Verbal
Thomas[79]	CC	CIDI	20; 20	36.0 ± 10.0	75.0	Yes	Yes	GN	Motor
van den Heuvel[80]	CC	SCID	15; 19	33.7 ± 9.7	46.7	No	No	PD	Motor
***C) Social phobia (SoP)***									
Amir[81]	CC	SCID	20; 20	35.3 ± 13.0	45.0	-	-	GP, SoP	Verbal
Boehme[82]	CC	SCID	16; 16	29.1 ± 9.8	16.67	No	Yes	SoP	Motor
Maidenberg[74]	CC	ADIS-R	15; 15	Range: 19–38	-	No	-	GN, GP, SoP	Verbal
***D) Specific phobia (SpP)***									
Britton[83]	CC	SCID	12; 12	25.2 ± 4.5	58.3	No	-	GN, SpP	Motor
***E) Multiple anxiety diagnoses***									
Andrews[84]	Crossover	SCID	11; 12	40.0 ± 7.0	45.5	No	Yes	ANX, GN, GP	Verbal
De Cort[67]	CC	DSM-IV	25; 30	36.0 ± 13.6	76.0	-	-	ANX, GN	Motor
Quero[85]	CC	DSM-IV	25; 25	29.0 ± 7.0	88.0	-	Yes	PD, SoP, GP	Motor

^A^ CBA = Controlled before and after study, CC = Case-control, RCT = Randomized Controlled Trial

^B^ DSM-III-R = Diagnostic Manual for DSM Disorders (3^rd^ Edition, revision), DSM-IV-TR = Diagnostic Manual of Mental Disorders (4^th^ Edition, text-revision), SCID = Structured Clinical Interview for DSM, ADIS-R = Anxiety Disorders Interview Schedule (revised), CIDI = Composite International Diagnostic Interview for DSM; G1 = Group 1, G2 = Group 2

^C^ GAD = Generalized anxiety disorder-relevant, GP = Generally positive, GN = Generally negative, PD = Panic disorder-relevant, SoP = Social phobia-relevant, SpP = Specific phobia-relevant; Rx = Prescriptions

#### Control groups

All clinical groups were compared to healthy control groups, without psychiatric or neurological disorder. The total number of participants in the healthy control groups was 1068 (260 participants in the PTSD studies; 266 participants in the MDD studies; 542 participants in the AD studies).

### Comorbidity and medication status

#### Comorbidity

Nineteen out of the 47 studies reported secondary diagnoses of other psychiatric and neurological disorders besides the primary diagnosis according to criteria of the DSM or ICD (Tables 1–3). For PTSD groups, 7 studies reported comorbid occurring diagnoses including AD (6 studies), MDD (6 studies), obsessive compulsive disorder (2 studies), personality disorder (2 studies), comorbid AD and MDD (1 study), eating disorder (1 study), somatoform disorder (1 study) and mild traumatic brain injury (1 study). For MDD groups, 2 studies reported comorbid diagnoses: AD (2 studies), dysthymic disorder (1 study), obsessive compulsive disorder (1 study), eating disorder (1 study), somatoform disorder (1 study), PTSD (1 study) and substance use disorder (1 study). For AD groups, 10 studies reported co-occurring disorders, including other AD (8 studies), MDD (6 studies), dysthymic disorder (2 studies), somatoform disorder (2 studies), eating disorder (1 study), personality disorder (1 study) and PTSD (1 study). Data on occurrence of comorbidity were insufficient to conduct moderator analyses to assess whether it has an impact on interference.

#### Medication status

In 22 out of the 47 selected studies, participants were reported to be undergoing pharmacotherapy (Tables 1–3). Individuals in the PTSD-groups received antidepressant (4 studies), anxiolytic (3 studies), first- or second-generation antipsychotics (2 study) and anticonvulsant (1 study) medication. One study reported that individuals in the PTSD-group were on current medication, but no specification on the type of drug was provided. In the MDD-groups, individuals received antidepressants (10 studies), anxiolytics (5 studies), first- or second-generation antipsychotics (3 studies). Individuals in the AD-groups were given antidepressants (7 studies), anxiolytics (5 studies), first- or second-generation antipsychotics (2 studies) or other non-specified medication (1 study). Any discrepancies in the number of clinical groups and studies reporting comorbid diagnoses or psychopharmacological treatment are related to individual studies assessing more than one clinical group (see for example Lim & Kim) [57], and clinical groups with individuals receiving customized (poly-) pharmacotherapy.

### Emotional Stroop interference

#### PTSD-groups

**Fig 2:**
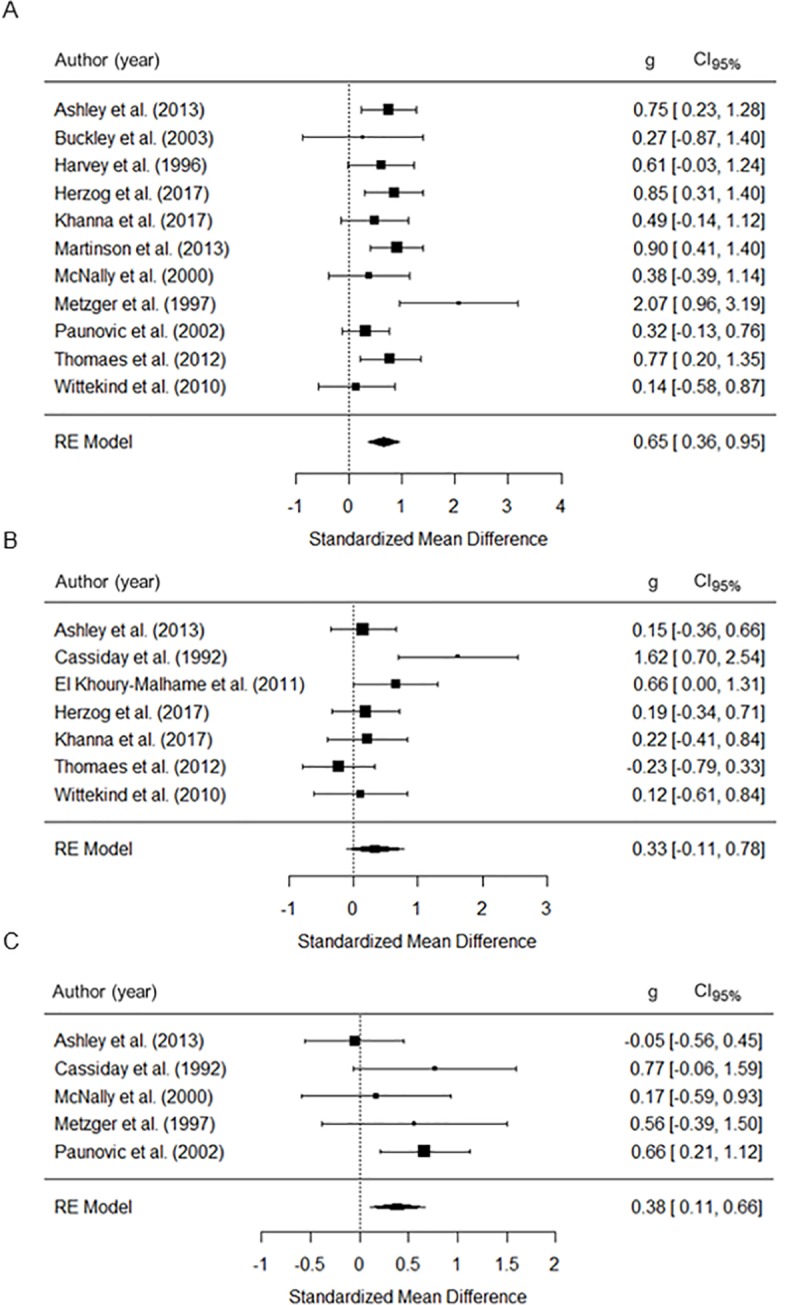
Effect sizes of emotional vs. neutral stimuli during emotional Stroop task performance for the PTSD-groups. (A) PTSD-specific words, (B) generally negative words, (C) generally positive words; RE = Random effects, g = Standardized mean-difference, CI_95%_ = 95% confidence interval.

As illustrated in **Fig 2**, the PTSD group, as compared to healthy control group, showed greater interference for PTSD-relevant and generally positive words (i.e. slowing of responses for PTSD-relevant and generally positive words as compared to neutral words). Effect sizes were strong on interference by PTSD-relevant words (*g* = .65, *p* < .001; Table 4) and moderate by generally positive words (*g* = .38, *p* < .01). Interference for generally negative words was not significant (*g* = .33, *p* = .139). Heterogeneity across studies was large for generally negative words (*Q’s p value =* .040, *I*^*2*^ *=* 71.53%). Cross-validation indicated that the interference remained non-significant for generally negative words when the meta-analysis was run multiple times, each time leaving out a different study (all *p-values* ≥ .068). Moderator analyses indicated that mean age of patients and female proportion did not influence attentional bias (all *p-values* ≥ .215, S3 Table). Response modality had a significant influence on effect sizes for interference by PTSD-relevant words only (p < .05). Effect sizes were larger for motor than verbal responses.

**Table 4:** Summary statistics of overall group comparisons for words with diagnosis-relevant, generally negative and generally positive stimulus valence in the emotional Stroop task.

Group comparison	Stimulus valence	N	g	g’s *p value*	SEM	g’s CI_95%_	Q’s *p value*	I^2^ (%)	I^2^ ‘s CI_95%_
PTSD vs. HC	PTSD-specific	11	.65	.000 (***)	.15	[.36, .95]	.209	57.91	[.00, 85.36]
	Generally negative	7	.33	.139	.23	[-.11, .78]	.040 (*)	71.53	[.00, 94.08]
	Generally positive	5	.38	.007 (**)	.14	[.11, .66]	.231	.00	[.00, 89.22]
MDD vs. HC	MDD-specific	8	.36	.005 (**)	.13	[.11, .62]	.147	37.70	[.00, 84.18]
	Generally negative	7	.58	.004 (**)	.20	[.18, .98]	.047 (*)	63.19	[.00, 92.57]
	Generally positive	9	.11	.293	.11	[-.10, .32]	.599	.00	[.00, 62.24]
AD vs. HC	AD-specific	24	.30	.000 (**)	.08	[.14, .45]	.057	35.28	[.00, 68.11]
	Generally negative	16	.32	.002 (**)	.10	[.12, .52]	.022 (*)	38.52	[1.50, 76.23]
	Generally positive	12	.05	.570	.08	[-.12, .21]	.588	.00	[.00, 58.57]

PTSD = Posttraumatic stress disorder; HC = Healthy controls; MDD = Major depressive disorder; AD = Anxiety disorder; N = Number of studies; g = standardized mean-difference; SEM = Standard error of the mean; CI_95%_ = 95% confidence interval; Q = Q-test of study heterogeneity.

(*) p < .05

(**) p < .01

(***) p < .001.

#### MDD-groups

**Fig 3:**
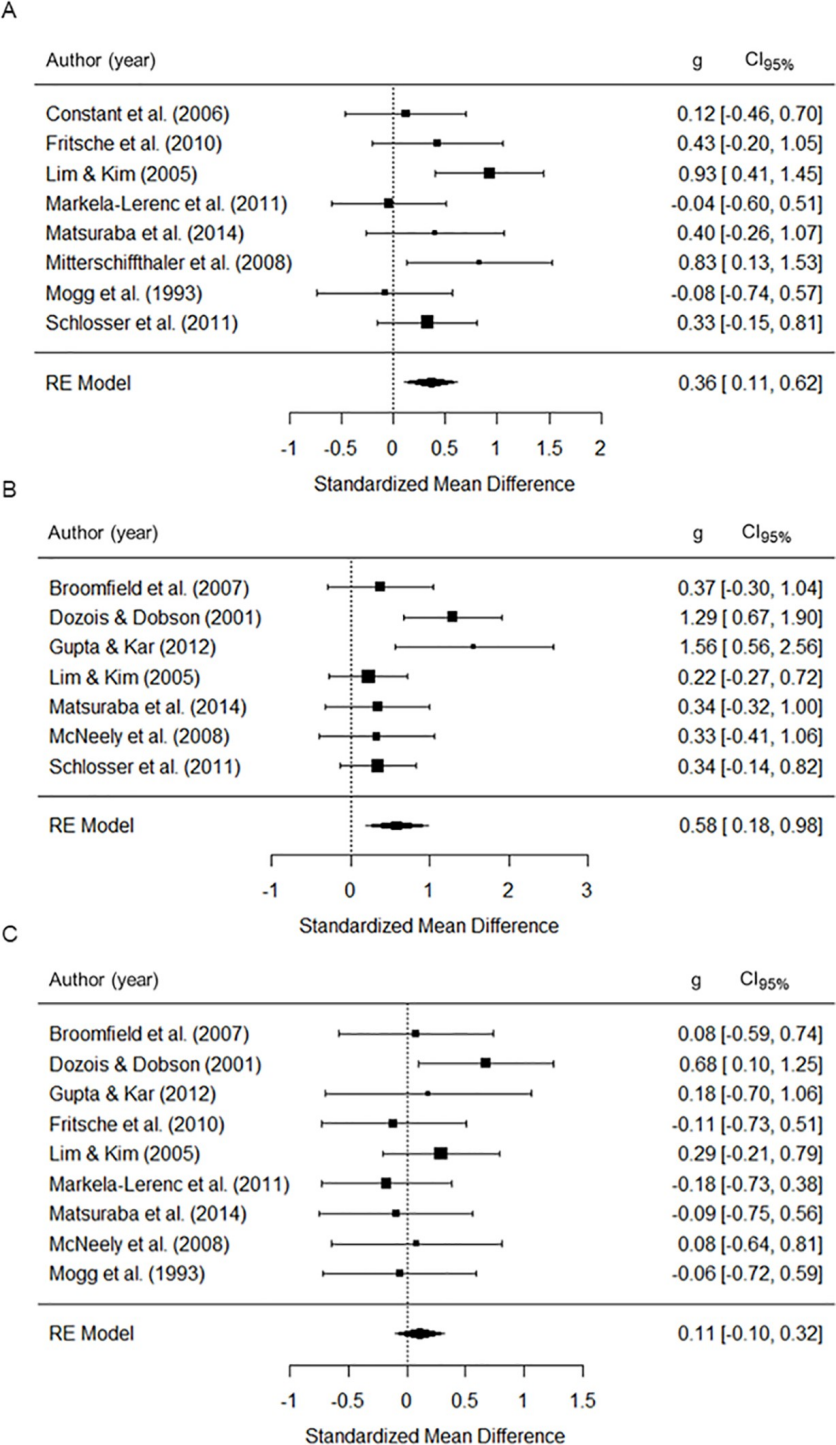
Effect sizes of emotional vs. neutral stimuli during emotional Stroop task performance for the MDD-groups. (A) MDD-specific words, (B) generally negative words, (C) generally positive words; RE = Random effects, g = Standardized mean-difference, CI_95%_ = 95% confidence interval.

The group composed of individuals with a primary diagnosis of MDD, as compared to healthy control group, displayed an attentional bias with moderate effect sizes for MDD-relevant words (*g* = .36, *p* < .01; Table 4; Fig 3) and generally negative words (*g* = .58, *p* < .01), but not for generally positive words (*g* = .11, *p* = .293). The Q-test of study heterogeneity was significant for generally negative words (*Q’s p value =* .047, *I*^*2*^ = 63.19%). The attentional bias for this stimulus valence remained significant when each study was removed one at a time in cross-validation analysis (all *p-values* ≤ 0.027). None of the moderator variables had a significant effect on the attentional bias in MDD-groups (all *p-values* ≥ 0.239, S3 Table).

#### AD-groups

**Fig 4:**
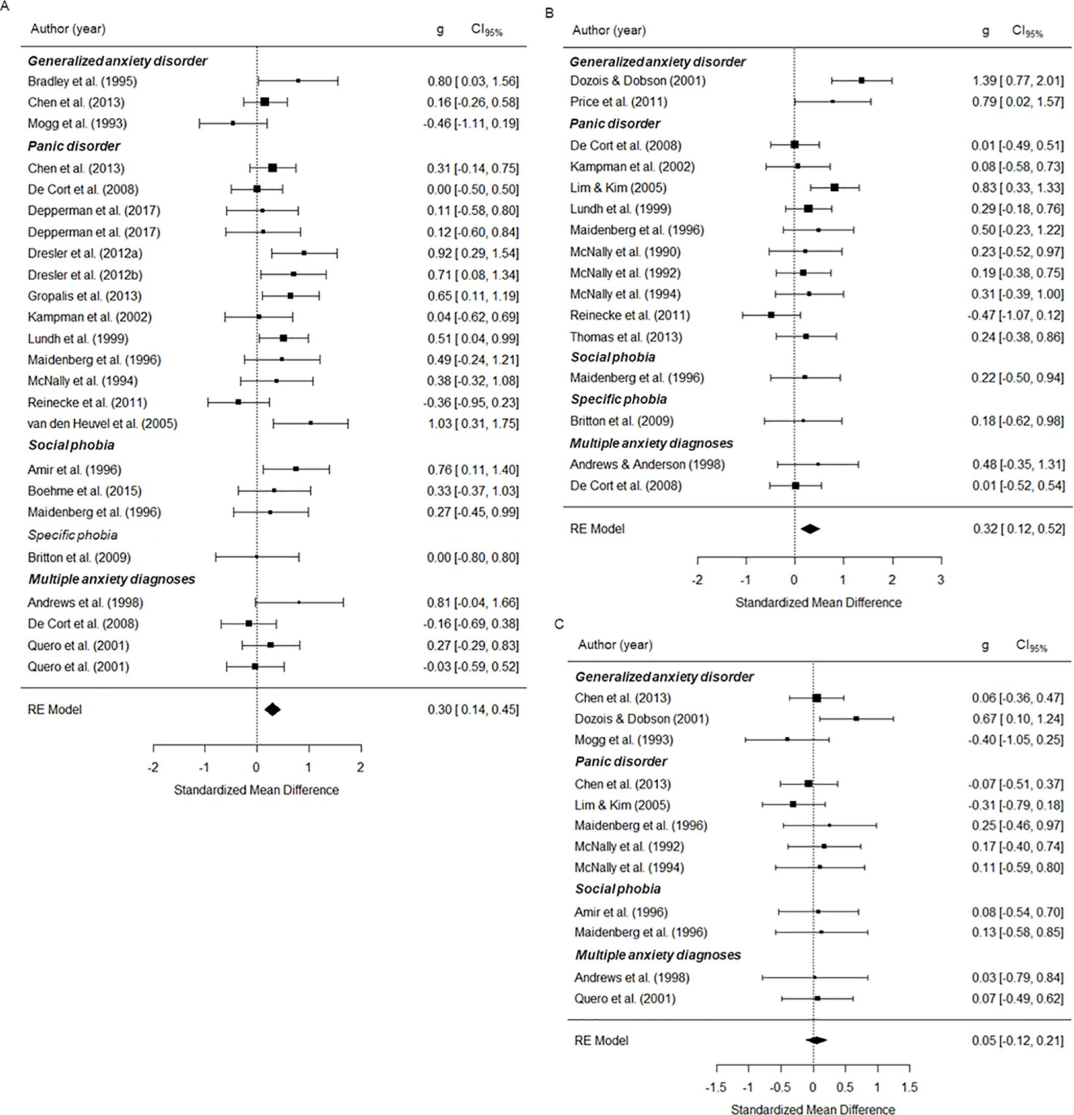
Effect sizes of emotional vs. neutral stimuli during emotional Stroop task performance for the AD-groups. (A) AD-specific words, (B) generally negative words, (C) generally positive words; Generalized anxiety disorder NOS = Generalized anxiety disorder—not otherwise specified, RE = Random effects, g = Standardized mean-difference, CI_95%_ = 95% confidence interval.

The group composed of individuals with a primary diagnosis of AD, as compared to healthy control group, showed an attentional bias with moderate effect sizes for AD-relevant words (*g* = .30, *p* < .001; Table 4; Fig 4) and generally negative words (*g* = .32, *p* < .01), but not for generally positive words (*g* = .05, *p* = .570). Between-study heterogeneity was large for generally negative words (*Q’s p value =* .022, *I*^*2*^ = 38.52). The attentional bias for generally negative words remained significant when we performed leave-one-out cross-validation (all *p-values* ≤ 0.009). None of the moderator variables had a significant effect on the attentional bias in AD-groups (all *p-values* ≥ 0.062, S3 Table).

### Risk of bias assessment

Twenty-five out for the 47 studies had a low risk of bias regarding incomplete outcome data, whereas the remaining 22 studies had an unclear risk of bias, due to insufficient reporting of exclusions or attrition. Thirty-three studies had a low risk of bias concerning the allocation concealment, one had a high risk of bias, and the remaining 13 studies had an unclear risk of bias. Regarding the selective reporting of outcomes, 40 studies had a low risk of bias, whereas 7 studies had an unclear risk of bias. All studies had a low risk of bias regarding blinding of participants and blinding of outcome assessments.

## Discussion

Attentional bias to emotionally loaded stimuli has been closely linked to PTSD symptom severity and is considered as one of the core cognitive deficits of PTSD. In this meta-analysis, we examined attentional bias in PTSD and in its most frequently concurrently occurring disorders, that is, MDD and AD. We assessed whether studies using the emotional Stroop task showed consensus findings regarding an attentional bias elicited by emotional stimuli in individuals diagnosed with PTSD, MDD and AD as compared to healthy individuals. The results indicate that PTSD, MDD and AD groups showed greater interference by diagnosis-relevant stimuli (i.e. slowing of responses for diagnosis-relevant stimuli in comparison to neutral stimuli) as compared to healthy control groups. Further, the PTSD-groups displayed greater interference by positive stimuli than the healthy groups, but this was not observed in the MDD- and AD-groups. Finally, the MDD- and AD-groups showed greater interference by generally negative stimuli as compared to the healthy groups, but this effect was not observed in the PTSD-groups.

Results of the current meta-analysis show that the PTSD-groups displayed greater interference by PTSD-relevant and positive stimuli, but not by generally negative stimuli, as compared to healthy controls. They support that patients are sensitive to stimuli related to their concern (see for example Bar Haim et al. or Williams et al.) [18,26]. This is of importance as such attentional bias to self-relevant stimuli may play a role in the maintenance of the disorders [88–90]. They are also similar to some extent to results from the meta-analysis of Cisler et al. [29] which reported greater interference for PTSD-relevant and threatening stimuli in the PTSD group as compared to the control group. Together, these findings support a hypoactive attentional control mechanism, rather than a hyperactive threat detection mechanism. The results also partially support the idea that a general-purpose defense mechanism can be automatically activated by given stimuli (e.g., PTSD-specific) which impacts seemingly irrelevant stimuli (e.g., positive stimuli) [91]. However, we cannot rule out that *positive* valence in this meta-analysis was not in some instances processed as PTSD-relevant or threatening (e.g., intimacy, love, sex, heal, brave, honor) by a given patient with PTSD would process this as diagnostic-relevant stimuli (e.g., rape or war). Future work should tailor attention bias tasks to individually relevant stimuli to elucidate this. Such interference by positive stimuli in PTSD was not observed in a previous meta-analysis [29]. The lack of significant group differences for interference by generally negative stimuli may suggest preserved attentional processing of negative emotional content unrelated to the traumatic event in PTSD. Similarly, Cisler et al. [29] found no difference between PTSD and healthy control groups regarding interference for generally negative stimuli. Interestingly, Clausen et al. [92] tested for potential correlations between PTSD symptoms and avoid-approach biases of emotional stimuli (happy, disgust, anger). They found a significant correlation indicating that greater symptom severity was associated with greater bias by happy faces. Together these results suggest that attentional processing of positively valenced stimuli might be more impacted than generally negative stimuli in PTSD. However, the present meta-analysis includes a small sample size with high heterogeneity for the generally negative stimuli which may also account for the lack of differences between the PTSD and healthy groups. As such, cross-validation analysis indicated a p-value of 0.068 when one study was removed [50].

In regards to MDD-groups and AD-groups, the findings show that attentional processing is impaired for diagnosis-relevant and negative stimuli, as interference was greater when compared to healthy controls. There were no significant group differences for positive stimuli. These data indicate that general attentional processing is to some extent intact in MDD and AD that fits with traditional models predicting that depression is associated with an attentional bias for mood-congruent stimuli [93,94] or self-relevant stimuli than general stimuli [26,88,90,95]. In regards to AD, the results are similar to those of a previous meta-analysis [18], which reported that patients with AD had greater interference by diagnostic-relevant and negative stimuli, but not by positive stimuli, at the emotional Stroop task. In regards to depression, a previous meta-analysis reported that patients clinically depressed (including dysthymia and minor depression) displayed greater interference by negative, positive, neutral stimuli at the emotional Stroop task, and even greater interference at the Classic Stroop task, when compared to healthy controls [30]. Effect sizes were greater for negative than positive stimuli and greater for positive than neutral stimuli. They also found greater interference in blocked than randomized design. Such impact of the design on interference was also observed in AD [18,26]. It is thus possible that interferences are more content-specific when using a randomized design than a blocked design (minimizing potential mood induction and not eliciting a generic system).

### The impact of age and sex on interference by emotional stimuli

We found that age and sex had no significant role in our moderator analyses for the PTSD, MDD and AD groups. Epp et al. [30] also reported no consistent patterns of moderating effects for age and sex in this meta-analysis in patients with depression. Age only had an impact on interference in healthy controls for incongruent versus control stimuli and sex only had an impact in depressed patients for neutral stimuli. In the current meta-analysis, response modality (verbal vs. button-press) had a significant impact for PTSD-relevant words when comparing the PTSD-groups with the healthy controls. Attentional bias was larger with motor than verbal responses. This difference may result from the modality pairing effect, which corresponds to the processing speed advantage for standard modality pairing (e.g. visual stimuli with motor response) as compared to non-standard modality pairing (e.g. visual stimuli with verbal response) [96]. Additional processes in the non-standard modality pairing condition may have diluted the interference effect. However, the lack of influence of the response modality for other stimulus valences in PTSD-groups as well as in MDD- and AD-groups advise caution about this finding.

Lack of data prevented us to use co-morbidity and pharmacological treatments as moderators. We cannot rule out that these factors had no impact on observed interference by emotional stimuli. Among studies included in the meta-analysis, some reported individuals’ current status of psychopharmacological treatment and diagnoses of comorbid disorders, but the majority did not. We cannot rule out that, simply because these covariates have not been reported, individuals were free of any exposure to psychotropic medications and other psychiatric or neurologic disorders that might have affected emotional Stroop task performance in PTSD. In studies comprising individuals with a primary diagnosis of MDD or AD, medication status was reported more frequently. However, groups were administered mono- or poly-pharmacotherapy, or received no medication at all, exacerbating the disentanglement of potential effects medical drug exposure might have had on behavioral outcomes in these cohorts. The same applies to the status of comorbidity in all three clinical groups of interest. When comorbidity was reported systematically, actual diagnoses were highly heterogeneous ranging from DSM axis I- and II-disorders [1] to neurological disorders. Moreover, our design was unsuited to investigate the effects of attentional bias to emotionally valent stimuli between PTSD and other disorders in explicitly comorbid cohorts (e.g., the impact of trauma-related or depression-related words in a group of individuals with a primary diagnosis of PTSD and comorbid MDD). This might come at the cost of potential interplaying effects on attentional bias when individuals are faced with more than a primary diagnosis of PTSD. However, assessing attentional bias in the emotional Stroop task for psychiatric disorders of interest separately might facilitate to disentangle effects that could, in turn, be responsible for attentional deficits in PTSD with frequent concurrent diagnoses. We also refrained from including a group of trauma-exposed, but PTSD-free individuals in our analysis, since the definition of such a control group was too heterogeneous across selected studies. Characterization of a trauma-exposed group might vary in subclinical scores on psychopathological questionnaires, PTSD in remission, as well as the mere presence of traumatic events during lifetime. It is hard to argue in favor of a trauma-exposed group if differentiation from a PTSD-diagnosed group (in terms of clinical features) in one direction and healthy controls (who might have experienced a traumatic event at least once in their life) in the other, if this border is rather transient than sharply defined. Lack of data prevented us to assess whether chronicity (e.g., time since trauma or diagnosis) was linked to greater interference, but previous work reported that greater severity of depression was linked to greater interference [30].

### Methodological considerations

This study has limitations to be considered. One limitation is that 32 studies included multiple effect sizes (either by including multiple conditions of the Stroop, multiple groups, or both). Correlations between effect sizes are likely to be stronger within than between studies, which can lead to partially redundant analyses and incorrect estimation of effects. Thus, our approach does not allow direct comparisons of effect sizes across task conditions. Future meta-analyses including multiple conditions should consider using a multivariate model [97]. Also, our number of group comparisons was considerably smaller than that of other meta-analyses assessing attentional bias in psychiatric disorders. This can be accounted for by our inclusion criteria (e.g., stroop task with words only supraliminal, unmasked stimuli), hence reducing variance in experimental designs across studies. On the one hand, this might account for the lack of attentional bias to negative words in PTSD (e.g., a smaller sample size); on the other hand, it might help to further understand the core mechanisms underlying attentional bias in PTSD and related psychiatric disorders. In addition, studies with smaller sample sizes might have introduced positive bias in some group comparisons, as indicated in the funnel plots (S1 Fig). For instance, it might be the case for generally negative words in PTSD- and MDD-groups, where we find studies with large effect sizes outside the funnel. By visual inspection of the corresponding forest plots (Figs 2–3), we identified two studies, that may have contributed to the heterogeneity observed in these group comparisons [43,56]. There was, however, no methodological argument to exclude these studies from our analyses. Moreover, these studies did not lead to increased heterogeneity across group comparisons on other emotionally loaded stimuli (i.e., words with generally positive valence). Cross-validation analyses also indicated that the results remained unchanged when we removed these studies. The considerable level of heterogeneity in effect sizes of studies comparing PTSD-groups and healthy controls on words with generally negative valence could partially explain the lack of statistical significance of this group comparison. Finally, some studies reported that the Stroop interference is reliable (see for example Ebersole et al.) [98], but other studies questioned it and suggest that individual differences play a significant role in such reliability [99,100]. The importance of stimuli choice is nicely discussed in the context of substance-related attentional bias by Field & Christiansen [101]. For instance, a given patient with alcohol use disorders might not respond the same to stimuli of heavy spirits than those of rosé wine. In sum, future work may consider using more than a single attentional bias task, which captures only a subset of processes [100], and add physiological measures such as an eye tracker to gather a more complete picture of bias to emotionally loaded stimuli that are relevant to symptoms of PTSD, MDD and AD.

## Conclusions

In sum, this review provides valuable synthesized results regarding an attentional bias for emotional stimuli in PTSD and its most frequently concurrent diagnoses of MDD and AD specifically elicited by the emotional Stroop task. However, it remains to be determined if the results presented here are specific to the emotional Stroop task or reflect a generally applicable cognitive deficit in PTSD and concurrent psychiatric disorders. We believe that additional studies are necessary to address the impact of concurrently occurring psychiatric disorders on the development and persistence of attentional bias in PTSD. Accumulated behavioral data might provide a clearer picture on underlying cognitive mechanisms that directly link to future treatment approaches (such as the ABM).

## Supporting information

S1 Fig Funnel plots for each diagnostic category, separately for words with diagnosis-relevant, generally negative and generally positive stimulus valence in the emotional Stroop task. (TIF)

S2 Fig Detailed search terms entered in databases for each clinical group. (TIF)

S1 Table PRISMA check-list. (DOCX)

S2 Table Assessment of risk of bias for each study included in the systematic review based on the Cochrane risk of bias tool. (DOCX)

S3 Table Results of moderator analyses for each clinical group with mean age of patients, percentage of female in clinical groups and response modality as moderator variables. (DOCX)
