# Supplementary figures and images for: Characterizing emotional Stroop interference in posttraumatic stress disorder, major depression and anxiety disorders: A systematic review and meta-analysis
Source: PLoS One. 2019 Apr 9;14(4):e0214998. doi: 10.1371/journal.pone.0214998 (PMC6456228; doi:10.1371/journal.pone.0214998)

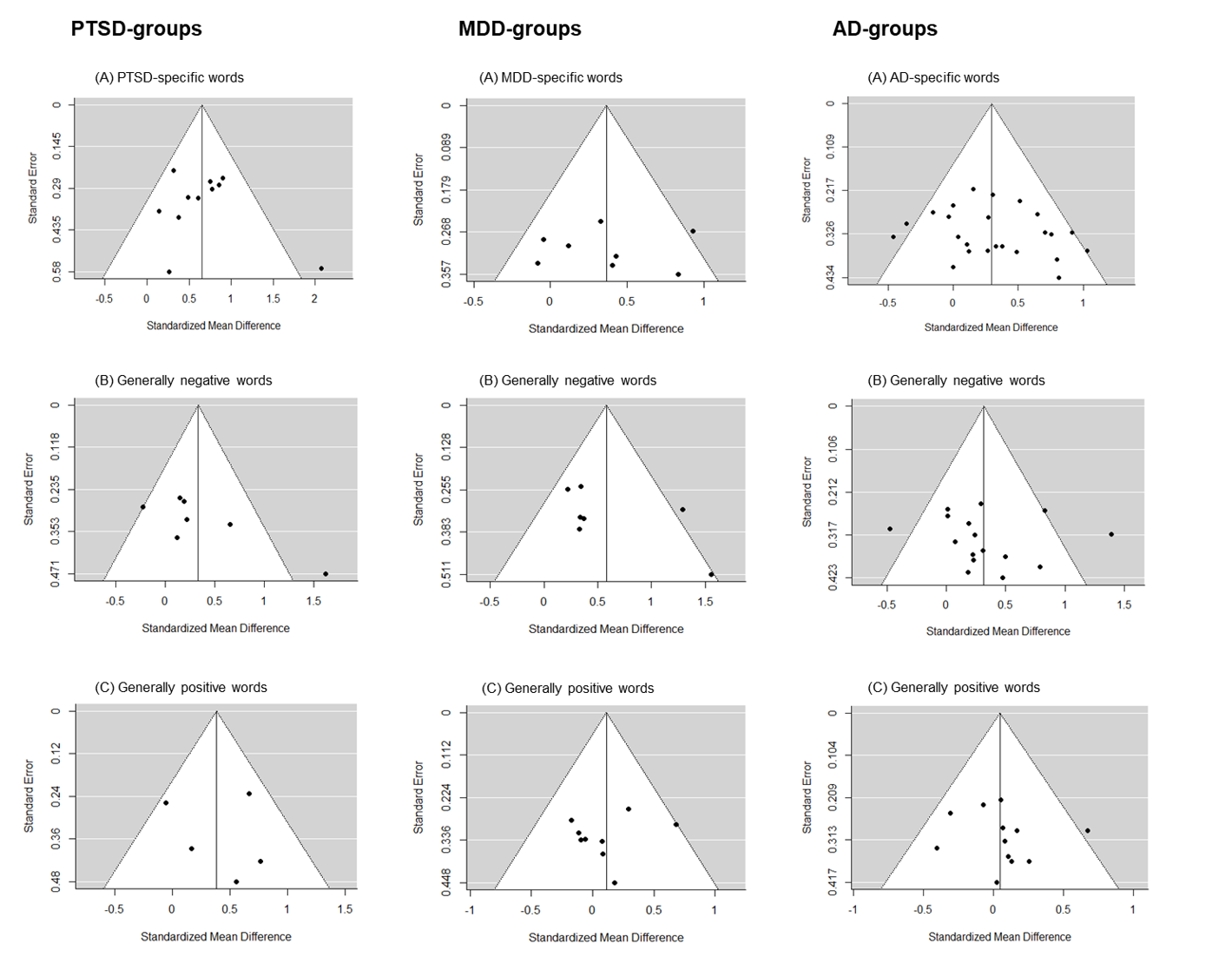

Supplement: S1 Fig — (TIF) [file pone.0214998.s001.tif]

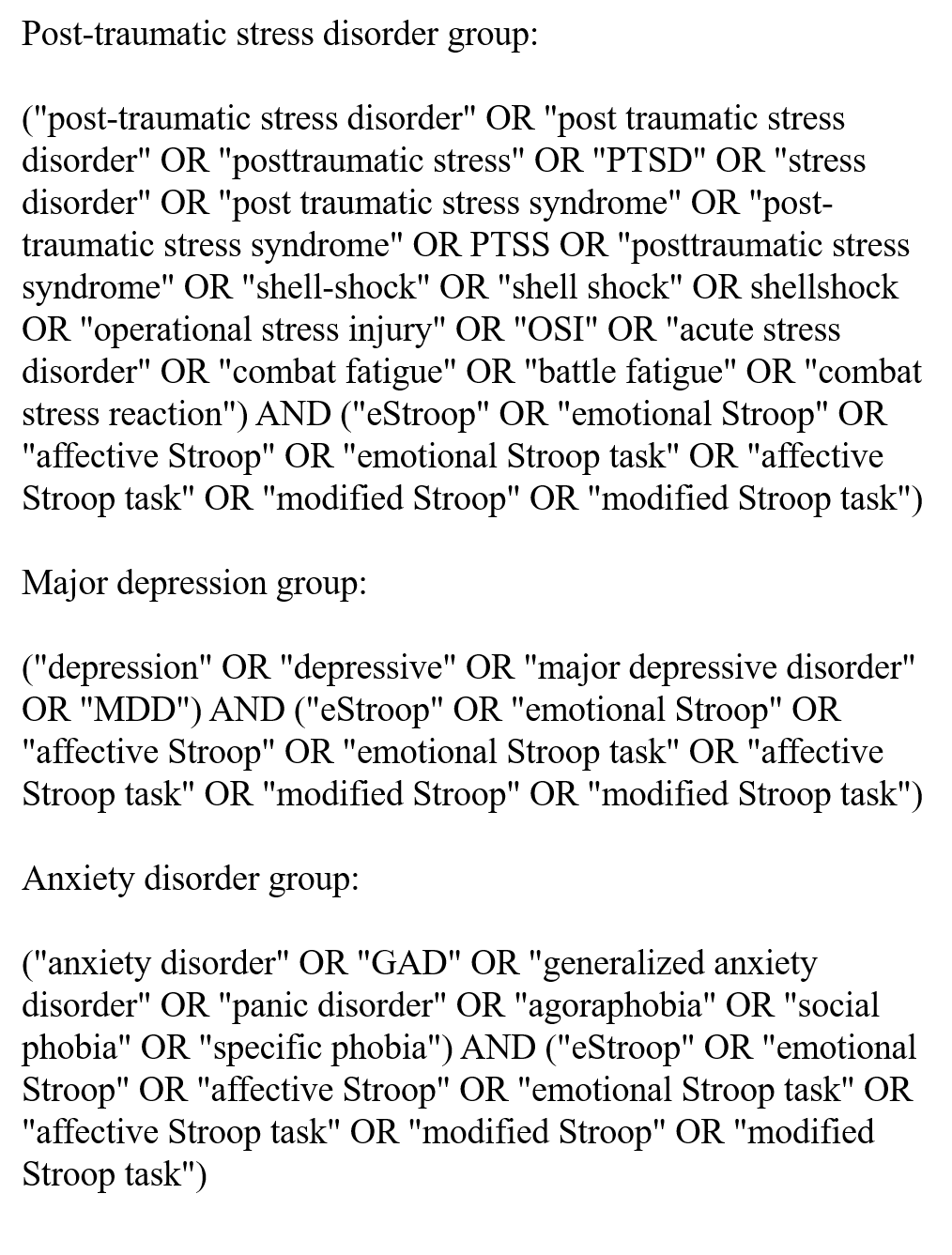

Supplement: S2 Fig — (TIF) [file pone.0214998.s002.tif]
