# Supplementary material for: Characterizing emotional Stroop interference in posttraumatic stress disorder, major depression and anxiety disorders: A systematic review and meta-analysis
Source: PLoS One. 2019 Apr 9;14(4):e0214998. doi: 10.1371/journal.pone.0214998 (PMC6456228; doi:10.1371/journal.pone.0214998)
Supplement: S2 Table — (DOCX) [file pone.0214998.s004.docx]

| **Author** |  | **Risk of bias assessment** | | | | | |
| --- | --- | --- | --- | --- | --- | --- | --- |
|  |  | **Allocation concealment** | **Blinding of participants and personnel** | **Blinding of outcome assessment** | **Incomplete outcome data** | **Selective reporting** | **Other sources of bias** |
| *Posttraumatic stress disorder (PTSD)* | | | | | | | |
| Ashley (5) |  | Low | Low | Low | Low | Unclear | Low |
| Buckley (42) |  | Low | Low | Low | Low | Low | Low |
| Cassiday et al. (43) |  | Low | Low | Low | Low | Unclear | Low |
| Harvey (44) |  | Unclear | Low | Low | Low | Low | Low |
| Herzog (45) |  | Low | Low | Low | Low | Low | Low |
| Khanna (46) |  | Low | Low | Low | Low | Low | Low |
| El Khoury-Malhame (6) |  | Low | Low | Low | Low | Unclear | Low |
| Martinson (47) |  | Unclear | Low | Low | Unclear | Low | Low |
| McNally (48) |  | Low | Low | Low | Unclear | Low | Low |
| Metzger (49) |  | Unclear | Low | Low | Unclear | Low | Low |
| Paunovic et al. (7) |  | Low | Low | Low | Unclear | Low | Low |
| Thomaes (50) |  | Low | Low | Low | Unclear | Low | Low |
| Wittekind (51) |  | Low | Low | Low | Unclear | Low | Low |
|  |  |  |  |  |  |  |  |
| *Major depressive disorder (MDD)* | | | | | | | |
| Broomfield (52) |  | Unclear | Low | Low | Low | Low | Low |
| Constant (53) |  | Low | Low | Low | Unclear | Low | Low |
| Dozois (54) |  | Unclear | Low | Low | Low | Low | Low |
| Fritzsche (55) |  | Low | Low | Low | Unclear | Low | Low |
| Gupta (56) |  | Unclear | Low | Low | Unclear | Low | Low |
| Lim (57) |  | Unclear | Low | Low | Unclear | Low | Low |
| Markela-Lerenc (58) |  | Low | Low | Low | Unclear | Low | Low |
| Matsubara (59) |  | Unclear | Low | Low | Unclear | Low | Low |
| McNeely (60) |  | Low | Low | Low | Low | Low | Low |
| Mitterschiffthaler (61) |  | Low | Low | Low | Unclear | Unclear | Low |
| Mogg (62) |  | Unclear | Low | Low | Low | Low | Low |
| Schlosser (63) |  | Low | Low | Low | Unclear | Low | Low |
|  |  |  |  |  |  |  |  |
| *Generalized anxiety disorder (GAD NOS)* | | | | | | | |
| Bradley (64) |  | Low | Low | Low | Low | Low | Low |
| Chen (65) |  | Low | Low | Low | Low | Low | Low |
| Dozois (54) |  | Unclear | Low | Low | Low | Low | Low |
| Mogg (62) |  | Unclear | Low | Low | Low | Low | Low |
| Price (66) |  | Low | Low | Low | Unclear | Low | Low |
|  |  |  |  |  |  |  |  |
| *Panic disorder (PD)* | | | | | | | |
| Chen (65) |  | Low | Low | Low | Low | Low | Low |
| De Cort (67) |  | Unclear | Low | Low | Unclear | Low | Low |
| Depperman (68) |  | Low | Low | Low | Low | Low | Low |
| Dresler (69) |  | Low | Low | Low | Low | Low | Low |
| Dresler (70) |  | Low | Low | Low | Low | Low | Low |
| Gropalis (71) |  | Low | Low | Low | Low | Unclear | Low |
| Kampman (72) |  | Low | Low | Low | Low | Low | Low |
| Lim (57) |  | Unclear | Low | Low | Unclear | Low | Low |
| Lundh (73) |  | Low | Low | Low | Low | Low | Low |
| Maidenberg (74) |  | Unclear | Low | Low | Low | Low | Low |
| McNally (75) |  | High | Low | Low | Low | Low | Low |
| McNally (76) |  | Low | Low | Low | Low | Low | Low |
| McNally (77) |  | Low | Low | Low | Low | Low | Low |
| Reinecke (78) |  | Low | Low | Low | Unclear | Unclear | Low |
| Thomas (79) |  | Low | Low | Low | Unclear | Unclear | Low |
| Van den Heuvel (80) |  | Low | Low | Low | Unclear | Low | Low |
|  |  |  |  |  |  |  |  |
| *Social phobia (SoP)* | | | | | | | |
| Amir (81) |  | Unclear | Low | Low | Unclear | Low | Low |
| Boehme (82) |  | Low | Low | Low | Unclear | Low | Low |
| Maidenberg (74) |  | Unclear | Low | Low | Low | Low | Low |
|  |  |  |  |  |  |  |  |
| *Specific phobia (SpP)* | | | | | | | |
| Britton (83) |  | Low | Low | Low | Unclear | Low | Low |
|  |  |  |  |  |  |  |  |
| *Multiple anxiety diagnoses* | | | | | | | |
| Andrews (84) |  | Low | Low | Low | Low | Low | Low |
| De Cort (67) |  | Unclear | Low | Low | Unclear | Low | Low |
| Quero (85) |  | Unclear | Low | Low | Low | Low | Low |
