# Supplementary material for: Characterizing emotional Stroop interference in posttraumatic stress disorder, major depression and anxiety disorders: A systematic review and meta-analysis
Source: PLoS One. 2019 Apr 9;14(4):e0214998. doi: 10.1371/journal.pone.0214998 (PMC6456228; doi:10.1371/journal.pone.0214998)
Supplement: S3 Table — (DOCX) [file pone.0214998.s005.docx]

| **Group comparison** | **Stimulus valence** | **Moderator** | **β** | **β’s CI_95%_** | **β’s**  ***p value*** | **SE** | **Q’s**  ***p value*** | **I^2^ (%)** |
| --- | --- | --- | --- | --- | --- | --- | --- | --- |
| PTSD vs. HC | PTSD-specific | Mean age | -0.0157 | [-0.0404, 0.0091] | 0.2150 | 0.0126 | 0.3649 | 55.89 |
|  |  | Percentage of female | 0.0011 | [-0.0075, 0.0097] | 0.8082 | 0.0044 | 0.1708 | 61.74 |
|  |  | Response modality | 0.4978 | [0.0763, 0.9192] | 0.0206(*) | 0.2150 | 0.6290 | 15.10 |
|  | Generally negative | Mean age | -0.0032 | [-0.0400, 0.0336] | 0.8638 | 0.0188 | 0.0219 (*) | 76.81 |
|  |  | Percentage of female | 0.0017 | [-0.0100, 0.0134] | 0.7797 | 0.0060 | 0.0220 (*) | 74.41 |
|  |  | Response modality | -0.2648 | [-1.1963, 0.6668] | 0.5775 | 0.4753 | 0.0279 (*) | 73.63 |
|  | Generally positive | Mean age | -0.0016 | [-0.0615, 0.0583] | 0.9573 | 0.0306 | 0.1332 | 14.31 |
|  |  | Percentage of female | 0.0046 | [-0.0033, 0.0125] | 0.2561 | 0.0040 | 0.2305 | 0.00 |
|  |  | Response modality | 0.1904 | [-0.8957, 1.2765] | 0.7311 | 0.5541 | 0.1414 | 30.77 |
| MDD vs. HC | MDD-specific | Mean age | -0.0043 | [-0.0797, 0.0710] | 0.9100 | 0.0384 | 0.0962 | 43.14 |
|  |  | Percentage of female | 0.0042 | [-0.0151, 0.0235] | 0.6689 | 0.0098 | 0.3800 | 16.85 |
|  |  | Response modality | -0.2340 | [-0.7679, 0.2999] | 0.3903 | 0.2724 | 0.1481 | 36.06 |
|  | Generally negative | Mean age | -0.0087 | [-0.0449, 0.0276] | 0.6396 | 0.0185 | 0.0294 (*) | 67.81 |
|  |  | Percentage of female | -0.0117 | [-0.0675, 0.0441] | 0.6811 | 0.0285 | 0.1700 | 64.31 |
|  |  | Response modality | -0.2039 | [-1.1249, 0.7172] | 0.6644 | 0.4699 | 0.0330 (*) | 67.22 |
|  | Generally positive | Mean age | -0.0027 | [-0.0232, 0.0178] | 0.7951 | 0.0105 | 0.4977 | 0.00 |
|  |  | Percentage of female | 0.0032 | [-0.0186, 0.0251] | 0.7721 | 0.0112 | 0.9828 | 0.00 |
|  |  | Response modality | -0.2512 | [-0.6689, 0.1665] | 0.2385 | 0.2131 | 0.6546 | 0.00 |
| AD vs. HC | AD-specific | Mean age | 0.0053 | [-0.0350, 0.0456] | 0.7973 | 0.0206 | 0.0175 (*) | 48.39 |
|  |  | Percentage of female | -0.0091 | [-0.0187, 0.0005] | 0.0623 | 0.0049 | 0.0653 | 35.43 |
|  |  | Response modality | 0.0010 | [-0.3277, 0.3297] | 0.9953 | 0.1677 | 0.0427 (*) | 38.32 |
|  | Generally negative | Mean age | 0.03 | [-0.004, 0.0600] | 0.0849 | 0.0152 | 0.0211 (*) | 46.13 |
|  |  | Percentage of female | 0.0010 | [-0.0130, 0.0150] | 0.8925 | 0.0071 | 0.4679 | 0.00 |
|  |  | Response modality | -0.1687 | [-0.6117, 0.2744] | 0.4556 | 0.2261 | 0.0224 (*) | 41.48 |
|  | Generally positive | Mean age | -0.0063 | [-0.0616, 0.0489] | 0.8218 | 0.0282 | 0.2646 | 19.39 |
|  |  | Percentage of female | 0.0009 | [-0.0105, 0.0124] | 0.8722 | 0.0059 | 0.9035 | 0.00 |
|  |  | Response modality | -0.0563 | [-0.3953, 0.2827] | 0.7446 | 0.1730 | 0.5079 | 0.00 |
